# Supplementary material for: Modeling glioblastoma heterogeneity as a dynamic network of cell states
Source: Mol Syst Biol. 2021 Sep 16;17(9):e10105. doi: 10.15252/msb.202010105 (PMC8444284; doi:10.15252/msb.202010105)
Supplement: Supplementary file 6 — Source Data for Figure 5 [file MSB-17-e10105-s004.zip › Figure5A_sourcedata/GSEA_3017/nosigdb_stateD.GseaPreranked.1622795010397/LARSSON_STATE_1.html]

Details for gene set LARSSON\_STATE\_1[GSEA]

|  || Dataset | state23017 |
| Phenotype | NoPhenotypeAvailable |
| Upregulated in class | na\_pos |
| GeneSet | LARSSON\_STATE\_1 |
| Enrichment Score (ES) | 0.23522644 |
| Normalized Enrichment Score (NES) | 1.5345228 |
| Nominal p-value | 0.032786883 |
| FDR q-value | 0.1604971 |
| FWER p-Value | 0.385 |
Table: GSEA Results Summary

  

Fig 1: Enrichment plot: LARSSON\_STATE\_1      
 Profile of the Running ES Score & Positions of GeneSet Members on the Rank Ordered List

  

| PROBE | GENE SYMBOL | GENE\_TITLE | RANK IN GENE LIST | RANK METRIC SCORE | RUNNING ES | CORE ENRICHMENT || 1 | PTTG1 |  |  | 14 | 0.490 | 0.0315 | Yes |
| 2 | PPP1R14B |  |  | 15 | 0.479 | 0.0728 | Yes |
| 3 | ARL4C |  |  | 24 | 0.422 | 0.1029 | Yes |
| 4 | SMS |  |  | 45 | 0.358 | 0.1184 | Yes |
| 5 | PHLDA1 |  |  | 59 | 0.336 | 0.1373 | Yes |
| 6 | LGALS1 |  |  | 83 | 0.308 | 0.1461 | Yes |
| 7 | LDHA |  |  | 92 | 0.297 | 0.1655 | Yes |
| 8 | TMEM158 |  |  | 120 | 0.275 | 0.1683 | Yes |
| 9 | RPS12 |  |  | 129 | 0.271 | 0.1855 | Yes |
| 10 | CCDC85B |  |  | 135 | 0.266 | 0.2046 | Yes |
| 11 | S100A16 |  |  | 142 | 0.262 | 0.2226 | Yes |
| 12 | RPL8 |  |  | 155 | 0.254 | 0.2352 | Yes |
| 13 | SPATS2L |  |  | 249 | -0.259 | 0.1857 | No |
| 14 | S100A13 |  |  | 427 | -0.276 | 0.0726 | No |
| 15 | PRDX6 |  |  | 475 | -0.282 | 0.0606 | No |
| 16 | DPYSL3 |  |  | 722 | -0.323 | -0.1017 | No |
| 17 | TMSB4X |  |  | 742 | -0.329 | -0.0881 | No |
| 18 | NQO1 |  |  | 756 | -0.333 | -0.0694 | No |
| 19 | ANXA2 |  |  | 777 | -0.338 | -0.0557 | No |
| 20 | PMEPA1 |  |  | 868 | -0.363 | -0.0940 | No |
| 21 | COTL1 |  |  | 907 | -0.376 | -0.0909 | No |
| 22 | TUBB4B |  |  | 916 | -0.380 | -0.0643 | No |
| 23 | DCBLD2 |  |  | 957 | -0.397 | -0.0610 | No |
| 24 | UPP1 |  |  | 1101 | -0.469 | -0.1312 | No |
| 25 | ANXA1 |  |  | 1221 | -0.623 | -0.1695 | No |
| 26 | SPP1 |  |  | 1277 | -0.787 | -0.1442 | No |
| 27 | CSRP2 |  |  | 1298 | -0.986 | -0.0746 | No |
| 28 | IGFBP5 |  |  | 1307 | -1.052 | 0.0101 | No |
Table: GSEA details [plain text format]

  

Fig 2: LARSSON\_STATE\_1: Random ES distribution      
 Gene set null distribution of ES for **LARSSON\_STATE\_1**
